# Correlation between Choroidal Neovascularization Shown by OCT Angiography and Choroidal Thickness in Patients with Chronic Central Serous Chorioretinopathy

**DOI:** 10.1155/2017/3048013

**Published:** 2017-10-04

**Authors:** Joanna Gołębiewska, Joanna Brydak-Godowska, Joanna Moneta-Wielgoś, Monika Turczyńska, Dariusz Kęcik, Wojciech Hautz

**Affiliations:** ^1^Department of Ophthalmology, The Children's Memorial Health Institute, Ul. Aleja Dzieci Polskich 20, Warsaw, Poland; ^2^Department of Ophthalmology, Medical University of Warsaw, Ul. Lindley'a 4, 02-005 Warsaw, Poland

## Abstract

**Purpose:**

To assess the occurrence of choroidal neovascularization (CNV) secondary to chronic central serous chorioretinopathy (CSCR) using optical coherence tomography angiography (OCTA) and correlate these findings with choroidal thickness (CT).

**Materials and Methods:**

This retrospective study included 25 consecutive patients (43 eyes), mean age 48.12 ± 7.8 years, diagnosed with persistent CSCR. All patients underwent a complete ophthalmic examination, fluorescein angiography (FA), indocyanine green angiography (ICGA), optical coherence tomography, and OCTA.

**Results:**

CNV was confirmed in 18.6% of eyes using FA and ICGA and in 25.6% of eyes using OCTA. All cases of CNV were associated with irregular retinal pigment epithelial detachment. CT was increased in the affected eyes (mean 491.05 ± 91.98), but there were no statistically significant correlations between CT and CNV and PED occurrence (*p* = 0.661 and *p* = 0.614, resp.) and between CT and duration of the disease (*p* = 0.940).

**Conclusions:**

OCTA detected CNV more frequently than other imaging modalities. CNV coexisted with irregular PED in all cases. CT was increased in eyes with chronic CSCR, but without any correlation with CNV occurrence; therefore, CT cannot be considered as a predictor of CNV occurrence. Further studies with a larger number of patients are needed to confirm these findings.

## 1. Introduction

Central serous chorioretinopathy (CSC or CSCR) is a frequently occurring chorioretinal disease, characterized by serous retinal detachment (SRD) in the macular area and/or focal detachments of an altered retinal pigment epithelium (RPE). CSCR affects mainly young to middle-aged people, predominantly men [1–3]. Corticosteroid use, hypercortisolism, type A personality, stress, hypertension, diabetes, genetic predisposition, alcoholism, and *Helicobacter pylori* infection have been associated with this disease [4–6]. With about a dozen hypotheses as to the CSCR etiology, the exact mechanism of the condition remains unknown [4–6]. The clinical diagnosis of acute CSCR form is quite evident, but the difficulties appear in older patients, with chronic CSCR, which may be complicated by choroidal neovascularization (CNV) and resembles age-related macular degeneration (AMD). Multimodal imaging, including spectral domain optical coherence tomography (SD-OCT), fluorescein angiography (FA), indocyanine green angiography (ICGA), and fundus autofluorescence (FAF), is used in the diagnostics and monitoring of CSCR, especially chronic forms [7, 8]. SD-OCT provides detailed images of the retina, and recently enhanced-depth imaging and swept-source technologies have given the opportunity for visualization of the choroid, improving the morphological analysis of choroidal vessels and choroidal thickness (CT) measurements. Accurate measurement of choroidal thickness *in vivo* is an important step in our understanding of CSCR pathophysiology and monitoring the course of the disease. Several studies showed increased choroidal thickness in patients with CSCR [9–14]. This finding is associated probably with hyperperfusion, which is one of the mechanisms responsible for CSCR. Optical coherence tomography angiography (OCTA) is an innovative, noninvasive tool, involving the detection and measurement of intravascular erythrocyte movement. OCTA delivers highly detailed, three-dimensional images of the entire microvasculature of the retina and choroid and helps assess retinal perfusion without intravenous dye injection. This new technique may be used in diagnosing different retinal vascular diseases, such as diabetic retinopathy, retinal vein occlusion, Coats' disease, and AMD [15–19]. OCTA has been also increasingly used in diagnosing patients with CSCR [20–22]. The latest studies focused on sensitivity and specificity of OCTA versus FA and ICGA in the detection of choroidal neovascularization complicating chronic CSCR and on the diagnostic discrepancies between these methods [23–28].

The aim of the study was to assess the occurrence of CNV secondary to chronic CSCR using OCTA and correlate these findings with choroidal thickness.

## 2. Material and Methods

25 consecutive Caucasian patients (43 eyes) diagnosed with persistent CSCR were enrolled in the retrospective study. All patients underwent a complete ophthalmic examination including a visual acuity test, intraocular pressure measurement, anterior segment examination, and dilated fundus biomicroscopy. Standard FA and ICGA with a Heidelberg retinal angiograph (HRA2, Heidelberg Engineering, Heidelberg, Germany) were performed in all subjects. The diagnosis of chronic CSCR was based on clinical history (visual impairment > 6 months) and FA characteristics. Exclusion criteria were diabetic retinopathy, previous ocular surgery, hereditary retinal dystrophies, vitreoretinal diseases, laser photocoagulation, pathologic myopia (defined as a spherical equivalent to −6 diopters or more), and a history of uveitis.

OCT and OCTA were performed using the commercially available RTVue XR Avanti with AngioVue (Optovue Inc., Fremont, CA, USA), which captures a 3 × 3 and 6 × 6 mm area centered on the fovea.

OCT B-scans were used to assess the retinal structure, the presence of subretinal and intraretinal fluid, retinal pigment epithelial detachment (PED), and other retinal pigment epithelium (RPE) pathologies (such as atrophy and hypo- and hyperpigmentation). Choroidal thickness, defined as the distance between the hyper-reflective line corresponding to the base of the RPE and the hyper-reflective line corresponding to the chorioscleral interface, was measured three times by two independent observers with manual calipers in the horizontal and vertical sections beneath the fovea, and average values were recorded and taken into analysis. Regarding the OCTA, we set the reference at the level of the choriocapillaris, defined as a 20 *μ*m band below the RPE-Bruch's membrane complex. Manual adjustment of the segmentation level was used to improve visualization. In OCTA images, we assessed the retinal and choroidal blood flow, mainly the presence of abnormal flow in a choroidal neovascularization network. Two experienced OCTA readers evaluated the images independently to determine morphological characteristics of the retina and choroid, to identify a CNV network, and to confirm the diagnosis. Only very high quality data (>60 signal strength index) were taken to analysis.

### 2.1. Statistical Analysis

Numerical traits were described by way of the measures of location—mean, median, and quartiles, and measures of dispersion—interquartile range, standard deviation, standard error of mean, 95% confidence interval, and minimum-to-maximum values. Discrete variables were presented as absolute numbers and frequencies (percentages).

Differences in the investigated measurements between two study groups, sectioned off according to a presence of choroidal neovascularization, were tested by using a one-way analysis of variance (ANOVA) without replication or the Mann–Whitney rank-sum test. Differences by occurrence of pigment epithelium detachment were verified by using a nested ANOVA. The normality of distribution and homogeneity of variances therefore had been appraised. The relationship between the central choroidal thickness and the disease duration time was estimated by using Pearson's product-moment correlation coefficient and also collated with robust regression estimates.

A level of *P* < 0.05 was considered statistically significant. All the statistical computations were carried out by means of Stata/Special Edition, release 14.2 (StataCorp LP, College Station, Texas, USA).

## 3. Results

The characteristics of the patients are summarized in Tables 1 and 2.

All retinal pathologies found in the affected eyes using FA, OCT, and OCTA techniques are summarized in Table 3.

**Figure 1:**
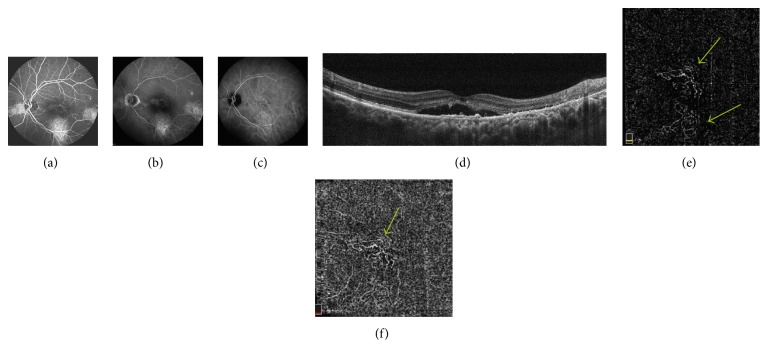
(a, b) FA—early phase (a) and late phase (b)—in the center of the macula, inferiorly, temporally to the fovea, and in the nasal part of the retina areas of hyperfluorescence due to RPE disorders—persistent epitheliopathy with subretinal fluid. (c) ICGA shows the corresponding FA areas of hyperfluorescence due to increased permeability of choroidal vessels and RPE disorders. (d) OCT B-scan shows the fovea involving subretinal fluid with shallow irregular PEDs in the center of macula, RPE irregularities temporally to the fovea. (e, f) OCTA at the level of the outer retina (e) and choriocapillaris (f) shows neovascular network (arrows).

**Figure 2:**
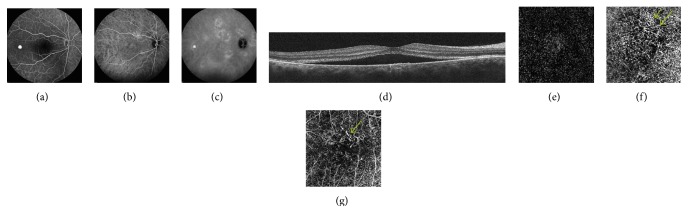
(a) FA—early phase—in the center of the macula hypofluorescence due to subretinal fluid, temporally to the fovea local hyperfluorescence due to small, serous PED. (b, c) ICGA—early phase (b) and late phase (c)—shows diffuse hyperfluorescence due to increased permeability of choroidal vessels, temporally to the fovea local hypofluorescence due to small, serous PED. (d) OCT B-scan shows the fovea involving subretinal fluid with shallow irregular PEDs in the center of macula. (e) OCTA shows the normal outer retina. (f) OCTA at the level of choriocapillaris shows the neovascular network (arrow). (g) OCTA—manual adjustment of segmentation level allowed to improve visualization of CNV (arrow).

CNV was detected by FA in 8 eyes (18.6%) and confirmed in ICGA in the same 8 eyes. OCTA revealed normal circulation in the superficial and deep retinal capillary plexuses in all eyes. A CNV network was detected in 11 eyes (25.6%) using OCTA, confirming the results of FA and ICGA in the abovementioned 8 eyes. In all cases of CNV, irregular PED was observed (Figures 1 and 2).

**Figure 3:**
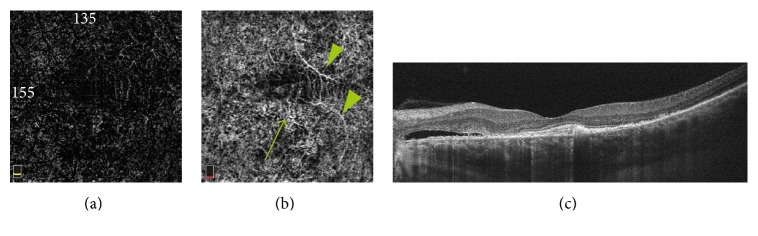
(a, b) OCTA at the level of the outer retina (a) and choriocapillaris (b) shows branches of CNV network (arrows) and artifacts due to superficial retinal vessel projection (arrow heads). (c) OCT B-scan, subretinal fluid nasally to the fovea, CNV in the center of the macula.

**Figure 4:**
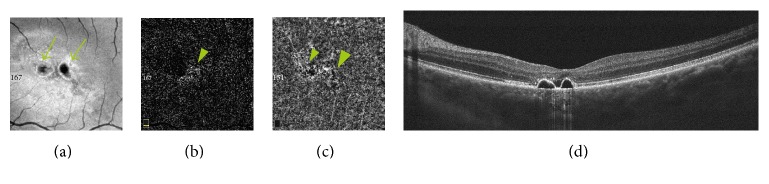
(a) En face OCT at the level of RPE reveals two hyporeflective areas due to serous PEDs (arrows) and hyper-reflective points due to RPE atrophy. (b, c) OCTA at the level of the outer retina (b) and choriocapillaris (c) shows decreased and increased flow signal areas due to PEDs and RPE disorders (arrows). (d) OCT B-scan—two regular, dome-shaped PEDs in the center of the macula with small amount of subretinal fluid.

Irrespective of the presence of CNV, OCTA showed abnormalities in choriocapillaris' blood flow in all affected eyes—focal or diffuse areas of increased or decreased flow signal. Areas of decreased flow signal were associated mainly with artifacts due to subretinal fluid, PEDs, and RPE migration. Focal or diffuse areas of increased flow signal were associated with RPE disorders, mainly RPE atrophy (Figures 3 and 4).

Choroidal thickness ranged from 260 to 636 *μ*m (mean 491.05 ± 91.98) in the affected eyes and from 270 to 577 *μ*m (mean 415.57 ± 113.57) in 7 fellow eyes; the difference was statistically significant (*p* = 0.049). CT did not correlate with disease duration, CNV, and PED occurrence (*p* = 0.940, *p* = 0.661, and *p* = 0.614, resp.) (Table 4).

## 4. Discussion

Central serous chorioretinopathy is a chronic macular disease involving choroidal vessel damage that causes fluid leakage, which leads to serous retinal detachment and visual deterioration. Vascular studies showed increased choroidal and reduced retinal blood flow in these patients [29, 30]. CNV secondary to chronic CSCR was reported in 2–10% of cases according to various data [31–33]. In those studies, FA was used in the diagnostics of CNV. In the recent years, several studies revealed superiority of OCTA versus FA and ICGA in the detection of CNV secondary to chronic CSCR. The authors emphasize diagnostic discrepancies between these methods, and although FA and ICGA are considered to be the “gold standards” in CSCR diagnostics, the use of OCTA enables much more precise detection of CNV—in 10–58% of eyes with chronic CSCR [23–28]. Our findings are consistent with the other authors; CNV was confirmed by FA and ICGA in 18.6% of the affected eyes and in 25.6% of eyes using OCTA. OCTA findings corresponded with those of FA and ICG in 100% of the cases, just as reported by Bonini Filho et al. [26]. We believe these discrepancies are due to the fact that patients with CSCR most commonly develop type 1 CNV and retinal abnormalities, such as subretinal fluid and epitheliopathy, and may have obscure type 1 CNV on FA images. OCTA showed a CNV network spreading to both the outer retina and the choriocapillaris or present only at the level of the choriocapillaris. In the 3 eyes with CNV detected only in OCTA, its network was visible only underneath the RPE. According to separate reports by de Carlo et al., Weng et al., and Hage et al., the irregular PEDs correlated with the presence of CNV in 19%, 11.4%, and 40.1% of cases, respectively [21, 23, 24]. Retinal PEDs are a common complication of CSCR, observed in 70–100% of cases; in the chronic form, they are usually irregular [25–28]. Irregular PED was presented in 51.2% of the eyes evaluated in our study, and all cases of CNV corresponded with shallow, irregular PEDs. It is important to realize that abnormal choroidal vascular pattern is not always associated with CNV. It may be caused by increased or decreased depth of OCT signal penetration due to such factors such as RPE irregularities or artifacts associated with overlapping superficial retinal vessels. Manual adjustment of the segmentation level often improves visualization and removes many artifacts. The results of our study are consistent with Constanzo et al., who reported that hyporeflective areas on OCTA images did not constitute evidence of decreased choroidal blood flow but appeared due to artifacts associated with subretinal fluid and PEDs, which may obscure the details of the underlying vasculature [27]. Many authors confirmed increased CT in patients with CSCR [9, 10]. It has also been shown that patients with unilateral CSCR have increased choroidal thickness in the unaffected eye, which suggests that elevated hydrostatic pressure in the choroidal vasculature occurs bilaterally [11]. Whether the choroid is thicker in chronic than in acute CSCR is still unclear, according to the available literature [12, 13]. The positive correlation between CT and risk of CSCR recurrences was also described [14]. Daruich et al. revealed increased choroidal thickness as a factor which influences episode duration in acute CSCR [34]. Based on previous studies, we might conclude that thicker choroid is a predictor of the worse course of the disease. The pathogenesis of CNV secondary to chronic CSCR is still unclear; therefore, we decided to check the correlation between CT and the occurrence of CNV. To the best of our knowledge, this is the first study on the association between CNV detected by OCTA and choroidal thickness in chronic CSCR. CT was increased in the evaluated group, both in the affected and fellow eyes, which is consistent with other reports [9–14, 34]. Our study did not reveal any statistically significant correlations between CT and the occurrence of CNV and type of PED. According to the results, we concluded that CT cannot be considered as a risk factor for CNV occurrence. Further researches with a larger number of patients are needed to confirm these findings.

## 5. Conclusions

OCTA detected CNV more frequently than other imaging modalities. CNV coexisted with irregular PED in all cases. CT was increased in the eyes with chronic CSCR but without any correlation with CNV occurrence; therefore, it cannot be considered as a predictor of future CNV.

## Figures and Tables

**Table 1:** Descriptive statistics for age (years), disease duration time (years), visual acuity (logMAR), and central choroidal thickness (*μ*m) in the studied patients (*n* = 25) or their eyes (*n* = 50).

Analyzed trait	Statistical parameter						
	M	Me	Q_1_–Q_3_ (IQR)	SD	SE	95% CI	Min.–max.
Age (years)	48.12	46	42–55 (13)	7.85	1.57	44.88–51.36	33–64
Disease duration time (years)	7.96	5	4–8 (4)	7.75	1.55	4.76–11.16	2–37
Best-corrected visual acuity (logMAR)	0.350	0.188	0.097–0.523 (0.426)	0.449	0.063	0.222–0.477	0.000–2.699
Central choroidal thickness (*μ*m)	480.48	489.50	428–557 (129)	97.63	13.81	452.73–508.23	260–636

Abbreviations: M: mean; Me: median; Q: quartiles; IQR: interquartile range; SD: standard deviation; SE: standard error; CI: confidence interval; logMAR: logarithm of the minimum angle of resolution.

**Table 2:** Descriptive statistics (frequencies) for discrete variables in the studied patients.

Patient characteristics	*n*	%
Patient gender (*n* = 25)		
(i) Females,	3	12.0
(ii) Males.	22	88.0
Number of affected eyes per patient (*n* = 25)		
(i) One,	7	28.0
(ii) Both..	18	72.0
Eyes in the study sample (*n* = 50)		
(i) Affected,	43	86.0
(ii) Nonaffected.	7	14.0

**Table 3:** Descriptive statistics (frequencies) for discrete variables in the studied patients affected eyes (*n* = 43).

Choroidal neovascularization detected in FA	8	18.6
Choroidal neovascularization detected in ICGA	8	18.6
Choroidal neovascularization detected in OCTA	11	25.6
Subretinal fluid	32	74.4
Pigment epithelium detachment		
(i) Absent,	13	30.2
(ii) Present, irregular,	22	51.2
(iii) Present, regular.	8	18.6
RPE atrophy	40	93.0

**Table 4:** Descriptive statistics for central choroidal thickness (*μ*m) in the studied patients and eyes by selected determinants.

Independent variable		Statistical parameter								
		M	Me	Q_1_–Q_3_ (IQR)	SD	SE	95% CI	Min.–max.	*P* value	
OCTA (*n* = 43)	CNV (*n* = 11)	476.64	538	305–584 (279)	136.11	41.04	385.19–568.08	260–636	=0.661	
	No CNV (*n* = 32)	496.00	494	442–554 (112)	73.40	12.97	469.54–522.46	320–620		

PED (*n* = 43)	Irregular (*n*–22)	504.05	526.50	450–584 (134)	107.35	22.89	456.45–551.64	260–636	=0.417	=0.614
	Regular (*n* = 8)	478.75	506.50	407.50–538.50 (131)	93.83	33.18	400.30–557.20	320–605		
	No PED (*n* = 13)	476.61	464	433–495 (62)	60.61	16.81	439.99–513.24	392–610		

Both eyes (*n* = 50)	Affected (*n* = 43)	491.05	495	433–564 (131)	91.98	14.03	462.74–519.35	260–636	=0.049	
	Nonaffected (*n* = 7)	415.57	428	290–528 (238)	113.57	42.92	310.54–520.60	270–577		

Abbreviations: M: mean; Me: median; Q: quartiles; IQR: interquartile range; SD: standard deviation; SE: standard error; CI: confidence interval; OCTA: optical coherence tomography angiography; CNV: choroidal neovascularization; PED: pigment epithelium detachment.
